# Virtual patients designed for training against medical error: Exploring the impact of decision-making on learner motivation

**DOI:** 10.1371/journal.pone.0215597

**Published:** 2019-04-23

**Authors:** Luke A. Woodham, Jonathan Round, Terese Stenfors, Aleksandra Bujacz, Klas Karlgren, Trupti Jivram, Viktor Riklefs, Ella Poulton, Terry Poulton

**Affiliations:** 1 Department of Learning, Informatics, Management and Ethics, Karolinska Institutet, Stockholm, Sweden; 2 Institute of Medical and Biomedical Education, St George’s, University of London, London, United Kingdom; 3 Karaganda Medical University, Karaganda, Kazakhstan; Universite de Bretagne Occidentale, FRANCE

## Abstract

**Objectives:**

Medical error is a significant cause of patient harms in clinical practice, but education and training are recognised as having a key role in minimising their incidence. The use of virtual patient (VP) activities targeting training in medical error allows learners to practice patient management in a safe environment. The inclusion of branched decision-making elements in the activities has the potential to drive additional generative cognitive processing and improved learning outcomes, but the increased cognitive load on learning risks negatively affecting learner motivation. The aim of this study is to better understand the impact that the inclusion of decision-making and inducing errors within the VP activities has on learner motivation.

**Methods:**

Using a repeated study design, over a period of six weeks we provided undergraduate medical students at six institutions in three countries with a series of six VPs written around errors in paediatric practice. Participants were divided into two groups and received either linearly structured VPs or ones that incorporated branched decision-making elements. Having completed all the VPs, each participant was asked to complete a survey designed to assess their motivation and learning strategies.

**Results:**

Our analysis showed that in general, there was no significant difference in learner motivation between those receiving the linear VPs and those who received branched decision-making VPs. The same results were generally reflected across all six institutions.

**Conclusions:**

The findings demonstrated that the inclusion of decision-making elements did not make a significant difference to undergraduate medical students’ motivation, perceived self-efficacy or adopted learning strategies. The length of the intervention was sufficient for learners to overcome any increased cognitive load associated with branched decision-making elements being included in VPs. Further work is required to establish any immediate impact within periods shorter than the length of our study or upon achieved learning outcomes.

## Introduction

### Medical error and education

Medical error has been widely identified as a key cause of preventable adverse events in clinical practice, with recent estimates indicating that it is the third leading cause of death in the US health system [1]. The impact of medical error was brought to the forefront of the debate on patient safety in 1999, when the US Institute of Medicine produced a report entitled “To err is human: building a safer health system”, as part of the Quality of Health Care in America project [2]. The findings of this report built upon previous studies and estimated that up to 98,000 people die each year in US hospitals as a result of medical errors by practitioners. This had a significant impact on the medical community both in the US and globally, and its recommendations helped to launch a significant drive to improve quality [3]. However, progress has been slow [4].

There has been an expanding recognition of the role to be played by education and training in targeting increased awareness of medical errors and minimising their incidence in diagnosis [5]. Learning by making errors and reflecting upon them can be a powerful educational tool, and simulation offers a particular opportunity for learners to make errors in a safe environment. When errors during such simulation exercises are approached in an educational environment that permits reflection upon negative emotions associated with error, learner awareness of the possibility of errors is raised, and allows learners to take responsibility for adapting their practice to avoid making the same or similar mistakes in future [6]. Indeed, while such educational approaches allow learners to make mistakes in a safe place, some educational experts contend that education should look further than that and seek to induce error in learners as a formative learning experience [7]. Eva acknowledges that this approach can be a challenging one for learners who are seeking positive affirmation of their knowledge through learning exercises, while also considering the powerful positive implications of learning from one’s mistakes, stating that “educators should be working to induce error in learners, leading them to short term pain for long term gain”.

The idea of inducing learners to experience “pain” as part of the learning process is a challenging one in an educational context. Although a great deal of focus in educational research is on providing a positive learner experience, it has been argued that learners are not always their own best judges of what is an effective learning experience [8]. Indeed, some have contended that emotions such as confusion on the part of the learner, providing they are effectively resolved by the end of the exercise, can be beneficial for the learning process [9]. This is not however to suggest that causing “pain” or “confusion” to the learner is desirable as a goal in itself, but that it can act a motivator to encourage greater engagement with learning materials through effortful problem-solving [10].

### The cognitive theory of multimedia learning

Mayer’s cognitive theory of multimedia learning [11] is a specialist application of Cognitive Load Theory [12], dealing with learning from words and pictures. They share a common triarchic theory of cognitive load, describing three processes that take place as part of learning [13]. The first, e*xtraneous processing*, is caused by poor instructional design providing information which is not relevant to the instructional objective i.e. managing *extraneous* cognitive load. *Essential processing* is the effort required for the learner to manage the *intrinsic* cognitive load and to cognitively represent or take in the given material. The level of essential processing is ultimately determined by the complexity of the material and is beyond the control of the instructional designer. Finally, *generative processing* is dedicated to managing the *germane* cognitive load which results from the learner seeking to make sense of the material, perhaps through reorganising or integrating the material within their existing knowledge. Generative processing is directly dependent upon the levels of motivation of the student towards the learning task [11,14]. Sound instructional design should seek to minimise unnecessary cognitive load in the form of extraneous processing [15], whilst fostering generative processing [14].

Worked examples of problems, as stepwise demonstrations of how to perform a task, have been shown to be effective at reducing extraneous processing [16]. Moreno and Mayer describe principles that can be adopted for effective multimedia learning, including the guided activity principle. This suggests that engaging students in interactive problem solving, and providing guidance in the form of feedback, can promote generative processing [17]. Generative processing can also be encouraged through self-explanation, in which learners are required to reflect upon instructional materials and develop their own explanations from them [18,19]. There is evidence from fields such as mathematics that generative processing can also be encouraged further by providing learners with examples of incorrect or erroneous working [20–22]. Within medical education, worked examples can be represented by patient cases. By providing patient cases to learners, combined with an instructional design that leverages emotions such as confusion to motivate greater learner engagement with problem-solving, feedback, and examples of poor practice or errors, focused resources can be delivered that will seek to target generative processing and improved learning outcomes.

### Virtual patients

Virtual patients (VPs) are interactive, online tools that place the learner within a simulated patient encounter [23], and are particularly well-suited to the teaching and development of skills related to clinical reasoning [24–29]. As a form of low to medium-fidelity simulation, VPs have become widely used in medical education, being used in a range of educational settings including small groups, lectures, self-directed learning and even for assessment [30–33], with and without supporting multimedia [34].

There are several different models for the design of VPs, but the use of a branched logic design allows learners to take decisions within the simulation and to understand the consequences of those decisions in a dynamically unfolding narrative [35]. The ability of branched VPs to incorporate decision-making elements within their structure has previously been leveraged to develop decision-problem-based learning, which uses VP resources as the basis for delivering small group teaching activities [27]. Such activities specifically aim to encourage learners to engage in generative processing through discussion and interaction, constructing mental models based upon prior knowledge in order to solve a problem [36].

Branched VP cases can give learners the opportunity to experience errors in patient management by providing sub-optimal options at key decision points. Such cases can deliver feedback on these choices via an unfolding narrative which explores the consequence of these sub-optimal decisions or errors, and are thus representative of worked examples of patient care in which erroneous working is modelled.

### Learner motivation

Applying Mayer’s model, VP cases seek to minimise extraneous processing by providing patient cases as worked examples. The inclusion of decision-making elements within these cases provides both interactivity and feedback, thus promoting generative processing according to the guided activity principle [17]. However, in applying these to the domain of medical error, the impact of learner motivation as a key driver for generative processing must also be considered.

A branching VP that incorporates decision-making elements may prompt learners to reflect upon their decisions and engage in self-evaluation of their own performance. Such a process may involve goal-setting and measuring their own performance against self-imposed standards for achievement. The attainability and proximity of these goals in relation to the current task is key when considering learner motivation; achievable, proximal goals closely related to a task are known to trigger heightened learner motivation to complete said task, while loose and unrelated goals serve only to demotivate effort [37].

Goal-setting can have a positive impact upon self-efficacy, meaning an individual’s belief in their own ability to perform a set task [38]. Self-efficacy also has an important role in determining learner motivation [39]. When evaluating one’s own performance against goals, perceived successes will raise self-efficacy while perceived failures will more likely serve to lower it [40]. Self-efficacy is a positive predictor of both learner effort and persistence as markers of motivation [41], and is linked to outcome expectancy. The outcome expectancy of a learner, that a particular course of action will achieve a specified outcome, is dependent upon the self-efficacy of the learner if it is to provide a motivational effect; if the learner lacks self-efficacy and doubts their ability to do what is needed, then they are unlikely to modify their behaviour since the expected outcome will be failure [39].

Learners who make errors in branched VPs may produce an emotional response to their perceived “failure” to meet their own standards, potentially resulting in a negative impact to their self-efficacy. As a consequence, we anticipate that the inclusion or otherwise of decision-making elements can be expected to greatly impact the balance, referred to by Eva [7], between the extent of the “long term gain” to learning resulting from increased generative processing, and the “short term pain” caused to learner self-efficacy and motivation. There is a lack of existing knowledge about the impact that the introduction of branched decision-making elements to VPs has on the motivation and self-efficacy of learners over time.

### Aims and research question

This study aimed to measure the impact upon motivation and learning strategies of using linear VP designs and VP designs that include branched decision-making by comparing the two designs and measuring the impact on motivation and self-efficacy. Our research question was whether the motivation and learning strategies of undergraduate medical students at participating institutions differed when given error virtual patient learning scenarios that contained or did not contain decision-making elements.

## Materials and methods

This study took place as part of the TAME (Training Against Medical Error) project, which aims to explore the use of VPs incorporating branched decision-making elements for developing awareness of medical error amongst undergraduate medical students [42]. The 3-year project, funded by the European Commission Erasmus+ programme, began in October 2015. The project partnership includes partners from 10 academic institutions across Europe and Central/South-East Asia.

### Study design

The study is a two-arm post-test cluster pseudo-randomised controlled trial. The primary independent variable is the design of the educational intervention; a suite of six virtual patients designed to train against medical error which incorporates branched decision-making elements, or the same virtual patients without branched decision-making elements.

The trial was replicated across six centres in three countries. The aim of replicating the study was to explore whether the findings were transferrable across different settings. We anticipated that local modifications to the interventions for language and institutional culture may impact the results, so determined that analysing the whole data set as one and ignoring the effect of the institution would not produce reliable findings. To address this, we analysed the results from each institution in isolation and treated each data set as part of a repeated study design. Results were then compared across institutions to assess the extent to which findings were transferrable. We chose to do this rather than include institution as a covariate in a single model, since each institution brings a complex mix of interacting factors (such as translation and adaptation of cases and instruments, and prevailing educational culture) that were not measured and would be unreliable as a single predictive factor. For completeness and as a point of comparison, we also ran the analysis over the whole aggregated dataset.

### Study population and participants

The study participants were drawn from undergraduate medical students at six medical schools from within the partnership of the TAME project; Karaganda State Medical University–Kazakhstan (KSMU), Astana Medical University–Kazakhstan (AMU), Bukovinian State Medical University–Ukraine (BSMU), Zaporozhye State Medical University–Ukraine (ZSMU), Hanoi Medical University—Vietnam (HMU) and Hue University of Medicine and Pharmacy–Vietnam (HUMP). Our inclusion criteria were that learners must be current undergraduate medical students at the participating institutions and enrolled in the Paediatric block of teaching as part of their studies during the period October 2016 to January 2017.

We integrated the intervention within the regular curriculum of the institutions, which already included regular small group teaching sessions. The acceptance of these sessions amongst staff and students has been well established [43,44]. Consequently, we chose to deliver the intervention to learners in their existing teaching groups and build upon this acceptance, allocating teaching groups as clusters to the two arms of the study, rather than doing so on an individual basis. Basic demographic details (age, gender) were collected from participants as part of the survey instrument to identify if there were any significant differences between the groups. Based upon the allocation of teaching groups, a total of 64 students were invited to participate at each of the institutions, with 32 allocated to each of the study arms. Study participants (learners) and tutors could not be blinded, as the nature of the educational intervention was apparent at the point the teaching sessions took place.

Since the intervention was implemented as part of the regular curriculum, learners were unable to opt out of the teaching sessions. However, the survey instrument included a consent statement at the start. Learners could choose to decline to provide consent if they wished to opt out of the study and not complete the survey. All required permits and approvals for the study were obtained, including those related to foreign researchers. Ethical approval for this study was provided by the Committee on Bioethics at the TAME project coordinators, Karaganda State Medical University (assigned no. 271). All project partners confirmed consent to participate in the study under the signed partnership agreement 2015-2944/001-002. Local approval for the project was granted at all countries and institutions following review from local bioethical committees, ministries and/or Heads of Curriculum Development and student experience/welfare.

### Intervention

**Fig 1:**
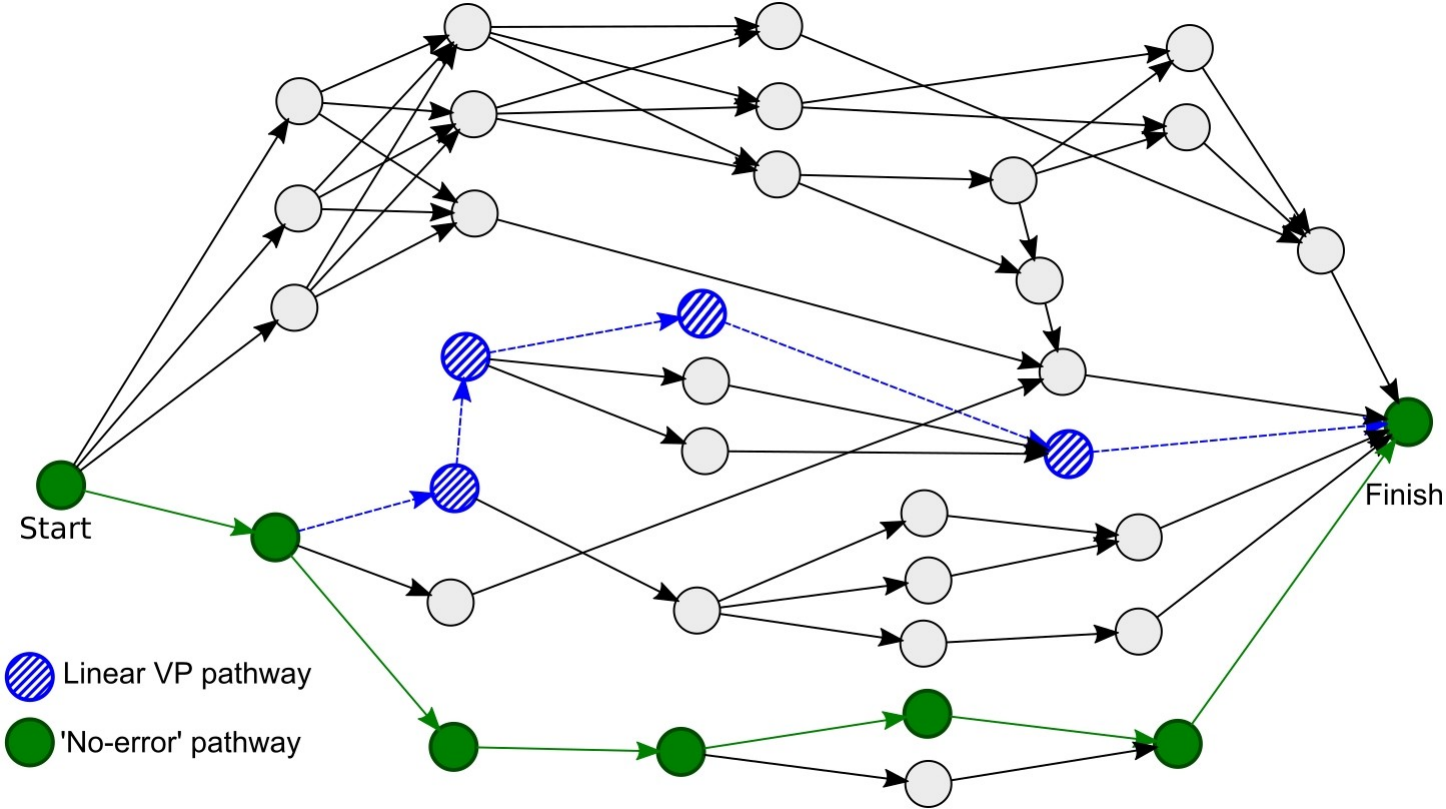
Case map showing the structure of a branching Error VP. The “ideal” pathway that represents good patient management decisions and includes no errors is shown in green. The linear variant of the VP is shown by the pathway that includes the blue diagonally shaded nodes.

Participants received six error VP cases, designed to cover a range of different errors and causes. These cases were provided to students over a period of around 6 weeks using the open-source OpenLabyrinth system, which is designed to support VPs with branched decision-making [45]. The time-period of six weeks was chosen to provide a prolonged exposure to the intervention that was broadly in-line with the length of a conventional learner placement in training, providing sufficient time to reflect upon a range of experiences. The cases given to the first group included branching logic at key trigger points, providing the possibility of making errors and exploring the resulting narrative. The diagram in Fig 1 gives an example of an error VP case map, showing the structure of the case as a network graph.

The second group received linear VPs which covered the same cases and topics, but with the key difference that they did not include branched decision-making elements, thus not providing the opportunity for learners to make decisions and subsequently to assume a sense of responsibility for any errors made. The content of the linear VPs represented a subset of the options available in the branched version, being a single pathway through the branched case, as shown in Fig 1. It should be noted that the linear VPs did not represent the ideal pathways through cases in which no errors are made; instead the case took learners through a series of errors without giving them the opportunity to take decisions and make errors themselves.

The VP interventions were written in English by a clinical paediatrician with expert experience of both creating VPs and medical error training. The English language cases were reviewed in discussion with colleagues from the e-learning team, all of whom have experience of creating VPs, and any necessary amendments were made. These amendments included typographic and grammar checks, but also included changes to the educational design of the VP to ensure consistency with and adherence to best-practice guidelines established in existing implementations [26,44].

The VPs were translated into the native/widely-spoken languages of the participant countries, with cultural adaptations made where required, while preserving the intended meaning and features. In all, the VPs were translated into Russian, Ukrainian and Vietnamese by members of the local project teams who were both fluent in the local language and had knowledge of the clinical culture and guidelines within that country. Having completed the translation and adaptation of the cases, a further review session was conducted with the original English case author. The adaptations made were discussed, in English, so that the case author was satisfied that the original decisions, and the associated errors that were afforded, still retained the educational value. Examples of adaptations that were required included changes to diseases that were extremely rare or unlikely to be recognised in a particular culture, such as dietary conditions caused by foods that are not widely available in the adapted setting.

Students in both groups used the VPs in a small group setting of 6–8 students facilitated by a tutor, in a standardised room layout with a computer workstation connected to a projector/smartboard, and a small group table. The group worked at a central table, while the VP was projected onto a smartboard using a PC workstation located at the front of the room. This room setup has been established through previous studies focusing on small group teaching [26,35], and is designed to promote discussion and minimise disruption to the group dynamic. Each session was designed to last for approximately three hours. At the end of each case teaching groups are prompted to reflect on the decisions that they made. If some of these decisions represented errors in practice, the group is asked to consider what factors may have underpinned these errors, based upon a taxonomy of clinical errors in practice [46].

### Instruments and outcome measures

Since seeking to encourage generative processing as a consequence of learner motivation and engagement with a learning activity is a goal of instructional design, it is important to have a means to describe and measure that engagement in order to evaluate a learning activity. One such instrument widely used in education is the Motivated Strategies for Learning Questionnaire (MSLQ) [47]. This survey instrument was designed to identify the key motivators and learning strategies used by undergraduate students. The instrument has been widely used and tested for validity in different settings [48], in global contexts [49] and specifically with medical students [50,51].

The instrument consists of fifteen different subscales of multiple ordered category scales measuring different aspects of the intended domain, which can be used either in isolation or together [47]. The motivation scales are based upon a general social-cognitive model of motivation covering three areas; expectancy (learners’ belief that they can accomplish a task), value (the reason for engaging in a task) and affect (learner emotions relating to a task) [52]. Those subscales related to motivation include, in the value domain: *Intrinsic Goal Orientation*, which refers to the extent in which a student is self-motivated to complete a task for the purpose of challenge or a desire for mastery of the subject; and *Task Value*, which is a measure of the student’s evaluation of the utility and importance of completing a task. In the expectancy domain, the scales include: *Control of Learning Beliefs*, which represents a student’s belief that completing the task will result in a positive outcome in their learning; and *Self-Efficacy for Learning and Performance*, a student’s appraisal of their own ability to perform a specified task. The learning strategies scales are based upon a general cognitive model of learning and information processing with three distinct areas; cognitive, metacognitive and resource management [52]. The learning strategies subscales include: *Critical Thinking* in the cognitive and metacognitive strategies domain, which is the student’s effort to engage in activities such as applying previous knowledge or problem-solving; and *Help Seeking* as an aspect of resource management, when learners recognise gaps in their own knowledge and seek assistance from their peers.

Following completion of the six error VP cases, we asked all participants in each group to complete a self-report survey instrument based upon a subset of statements from the Motivated Strategies for Learning Questionnaire (MSLQ). Each item of the MSLQ requires participants to provide a rating on a seven-point ordered category scale, ranging from “not at all true of me” to “very true of me”. The overall scores for each subscale of the MSLQ are calculated by taking the mean of the items for that subscale. Some items are negatively worded, and the responses for these items must be reversed before being numerically coded [47].

We adapted the original instrument to be applicable to VPs where required, since the original was designed to be used in reference to a class or module. Most of the adaptations generally involved replacing references to “class” with references to “cases”, although some modifications were more substantial. Of the 15 subscales in the MSLQ, we determined that 6 of the subscales were applicable to this context and relevant to this study; of the remaining subscales that were excluded, the bulk related to assessments or external study practices (including time management and peer support) none of which were relevant to the non-assessed, self-contained learning interventions provided to the study participants. The scales selected included elements from both the Motivation and Learning Strategies domains, and were Intrinsic Goal Orientation, Task Value, Control of Learning Beliefs, Self-Efficacy for Learning and Performance, Critical Thinking and Help Seeking. No items were removed from these subscales prior to data collection. These scales most closely related to our hypothesis, measuring the motivation and extent to which the learners were engaging in activities that foster generative processing. An additional single item which related to the subscale describing the elaboration strategies of learners was included in the original survey but removed from analysis on the grounds of reliability, since no other items from that subscale were included with which to measure internal consistency. The full list of statements and scales included is provided in Table 1. A printed, English version of the survey instrument can be seen in S1 File.

**Table 1:** List of subscales and statements used from the MSLQ.

Subscale	Statement
Intrinsic Goal Orientation	When working on a patient scenario, I like when it really challenges me so I can learn new things.
	The most satisfying thing for me in these cases was trying to understand the content as thoroughly as possible.
	When I was choosing options in these cases, I sometimes chose the option that I felt I could learn from even if I thought it was incorrect.
	When working on a patient scenario, I prefer it to cover material that arouses my curiosity, even if it is difficult to learn.
Task Value	Understanding the subject matter of these cases is very important to me.
	I like the subject matter of these cases.
	I think I will be able to use what I learnt in these scenarios in other areas of my training.
	It is important for me to learn the material in these scenarios
	I think the material in these cases is useful for me to learn.
	I am very interested in the content area of these scenarios.
Control of Learning Beliefs	If I study in appropriate ways, then I will be able to learn the material in these cases.
	If I don't understand the material in these cases, it is because I didn't try hard enough.
	It is my own fault if I don't learn the material in these cases.
	If I try hard enough, then I will understand the material in these cases.
Self-efficacy for Learning and Performance	I'm certain I can understand the most difficult material presented in the readings for these cases.
	I'm certain I can master the skills being taught in these cases.
	I expect to do well in these cases.
	I'm confident I can understand the most complex material presented in these cases.
	Considering the difficulty of the cases, the tutor, and my skills, I think I will do well in these sort of scenarios.
	If assessed, I'm confident I could do an excellent job on the assignments and tests relating to these cases.
	If assessed, I believe I would receive an excellent grade in these cases.
	I'm confident I can understand the basic concepts taught in these cases.
Critical Thinking	Whenever I read an assertion or conclusion in the scenarios I thought about possible alternatives.
	I often found myself questioning things I read in the scenarios to decide if I found them convincing.
	I treated the cases as a starting point and tried to develop my own ideas about it.
	When a theory, interpretation, or conclusion was presented in the scenarios I tried to decide if there was good supporting evidence.
	I tried to play around with ideas of my own related to what I was learning through the scenarios.
Help Seeking	I asked the tutor to clarify concepts I didn't understand well.
	When I couldn't understand the material in the scenarios, I asked another student in the class for help.
	I tried to identify other students in the class whom I could ask for help if necessary.
	Even if I was struggling with the scenarios, I tried to do the work on my own, without help from anyone. (Reversed)

Having finalised the survey instrument in English, a native language speaker from the project team in each country translated the survey into the required languages; Russian, Ukrainian and Vietnamese. Translations were checked by a second native language speaker and translated back into English to confirm the accuracy of the original translation. Where meanings had seen to be changed, these were modified and corrected in the final translation by negotiation between the translators.

An online version of each translation of the survey instrument was created using the online tool SurveyMonkey [53]. Depending upon their practical needs and the feasibility of their approaches, the institutional partners were able to choose whether they distributed the surveys to participant students electronically using a specified collector web address through SurveyMonkey or using printed paper copies. If using paper completion, it was the responsibility of the partner institution to subsequently use the paper copies to enter the responses into SurveyMonkey.

### Data analysis

We analysed all data using the statistical package R [54].

Due to the new context in which the survey was being used, and the adaptations that had been made to some of the ordered category scales, we initially conducted a reliability analysis on the survey instrument based upon the data received to assess its validity in this new setting. For each translation of the survey we calculated Cronbach’s alphas, corrected item-total correlation and correlation matrices for each subscale to measure its internal consistency [55]. Where an item was shown to lack internal consistency i.e. the alpha of the subscale would be increased by removing that item, items would be removed. However, for the purposes of accurate comparison between sites, we determined that the same items should be retained for each translation. Accordingly, an item was removed from the subscale only if the analysis suggested that it should be removed from two or more of the three translations; if so, it would be removed from all three translations.

Similarly, we reviewed the alpha for each subscale to identify if the internal consistency was sufficiently high to indicate that it was a reliable measure. A guiding threshold of 0.7 was viewed as suitable for retention but was not applied rigidly. For example, if the subscale still contained multiple items and the alpha was close to the threshold this was taken to indicate that the measurement was worthwhile retaining.

Two-tailed unpaired student t-tests were used to determine the significance of differences between the groups using branched and linear VP cases, and plots were created to show mean values and 95% confidence intervals.

## Results

In total, across all institutions, 346 out of a possible 384 students completed the survey instrument, giving a response rate of 90.10%. Response rates at the different institutions varied between 81.25% and 100%, with the number of students who declined to participate and complete the survey at each site varying between 0 and 12. The average age of respondents varied between 21.00 years old and 23.75 years old. All students were at the same stage of training, enrolled in the paediatric block which took place in the clinical years of undergraduate medical training. In general, there were a considerably higher number of female participants at each institution, but this was approximately equally reflected in both arms of the study and was also true in all institutions. The breakdown of participant numbers at each site, along with descriptive statistics for age broken down by gender, is shown in Table 2. The full dataset of responses can be found in S1 Dataset.

**Table 2:** Response rates and population description at each institution.

	Total		By Study Arm					By Gender			
Institution	No. Responses	Response rate (%)	Study Arm	n	Response Rate (%)	Mean Age	SD Age	Gender	n	Mean Age	SD Age
BSMU	58	90.63	Branched	29	90.63	22.72	0.75	F	21	22.67	0.57
								M	8	22.88	1.13
								N/A	0	-	-
			Linear	29	90.63	23.14	1.35	F	20	22.90	1.29
								M	8	23.75	1.49
								N/A	1	23.00	-
HMU	56	87.50	Branched	26	81.25	22.04	0.45	F	15	21.93	0.26
								M	11	22.18	0.60
								N/A	0	-	-
			Linear	30	93.75	22.60	2.75	F	19	22.89	3.45
								M	11	22.09	0.30
								N/A	0	-	-
HUMP	55	85.94	Branched	28	87.50	22.00	0.00	F	23	22.00	0.00
								M	5	22.00	0.00
								N/A	0	-	-
			Linear	27	84.38	22.00	0.00	F	18	22.00	0.00
								M	9	22.00	0.00
								N/A	0	-	-
AMU	58	90.63	Branched	29	90.63	21.00	0.38	F	20	20.95	0.39
								M	9	21.11	0.33
								N/A	0	-	-
			Linear	29	90.63	20.96	0.19	F	21	20.95	0.22
								M	8	21.00	0.00
								N/A	0	-	-
KSMU	52	81.25	Branched	25	78.13	20.96	0.93	F	17	20.89	0.78
								M	8	21.13	1.25
								N/A	0	-	-
			Linear	27	84.38	21.63	1.27	F	19	21.37	1.01
								M	8	22.25	1.67
								N/A	0	-	-
ZSMU	64	100.00	Branched	32	100.00	22.81	3.63	F	22	22.54	3.90
								M	10	23.40	3.06
								N/A	0	-	-
			Linear	32	100.00	22.50	0.58	F	26	22.33	0.58
								M	6	23.00	-
								N/A	0	-	-
Not given	3	-	Branched	1	-	22.00	-	F	1	22.00	-
								M	0	-	-
								N/A	0	-	-
			Linear	2	-	22.00	0.00	F	2	22.00	0.00
								M	0	-	-
								N/A	0	-	-
Total	346	90.10		346					346		

The reliability analysis generally indicated that the alphas for each subscale were above the accepted 0.7 threshold, indicating good internal consistency, with two exceptions caused by single items. The item “When I was choosing options in these cases, I sometimes chose the option that I felt I could learn from even if I thought it was incorrect” had an item-total correlation of between 0.32 and 0.43 with the other items in the Intrinsic Goal Orientation subscale. Likewise, the reverse coded item “Even if I was struggling with the scenarios, I tried to do the work on my own, without help from anyone” had a negative item-total correlation with the other items in the Help Seeking subscale. Both these items were dropped and the alphas were recalculated, then ranging from 0.72 to 0.91, with only the Help Seeking scale lower (alpha between 0.6 and 0.68). Since the alpha for the Help Seeking scale was only marginally below the threshold, it was deemed to have value in interpreting the results and retained. All other subscales were retained as demonstrating acceptable internal consistency following the second iteration of the analysis.

**Fig 2:**
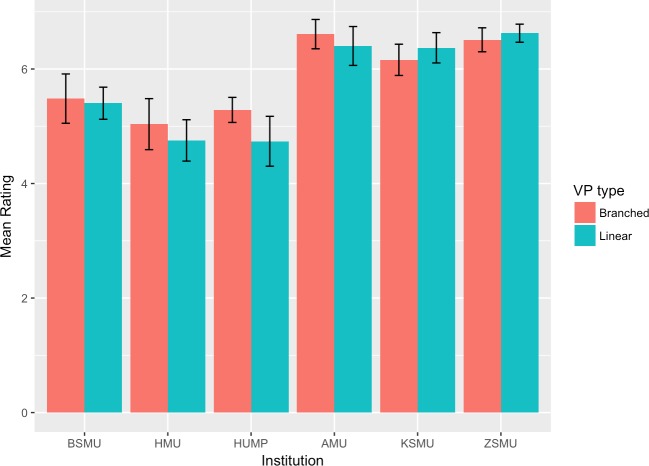
Intrinsic goal orientation mean ratings.

**Fig 3:**
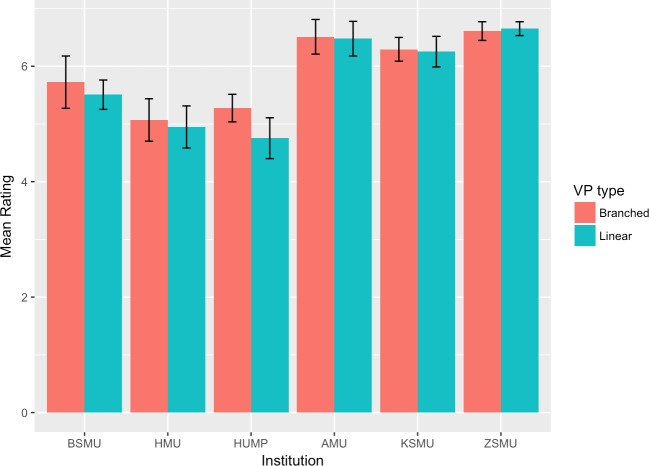
Task value mean ratings.

**Fig 4:**
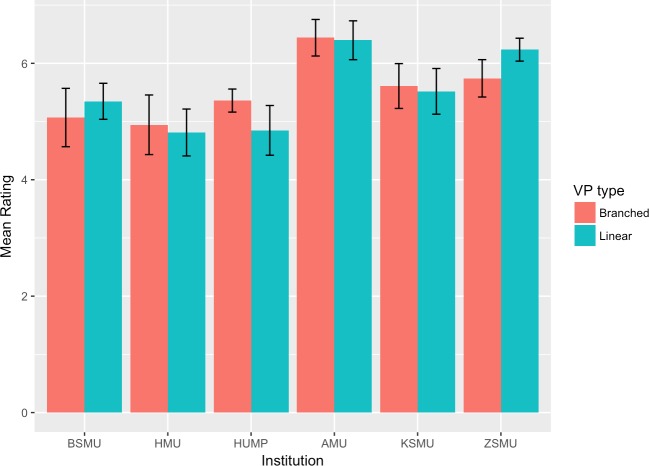
Control of learning beliefs mean ratings.

**Fig 5:**
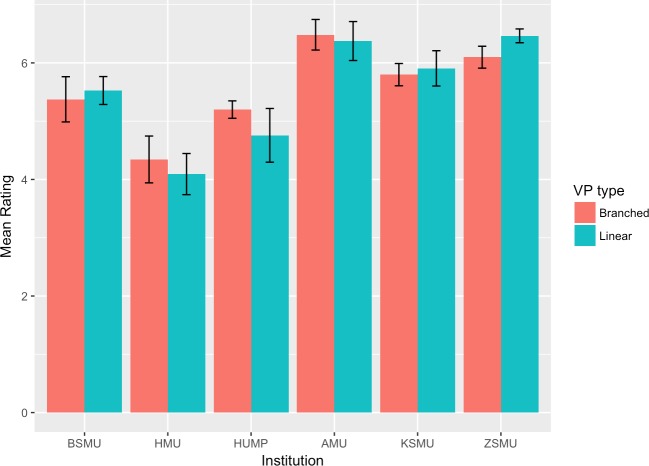
Self-Efficacy for learning and performance mean ratings.

**Fig 6:**
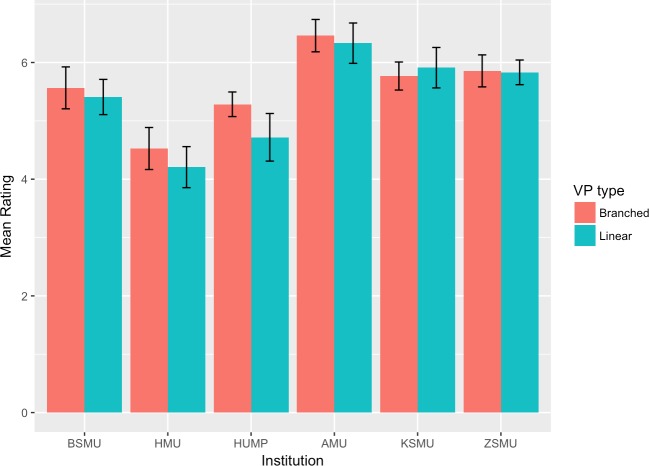
Critical thinking mean ratings.

**Fig 7:**
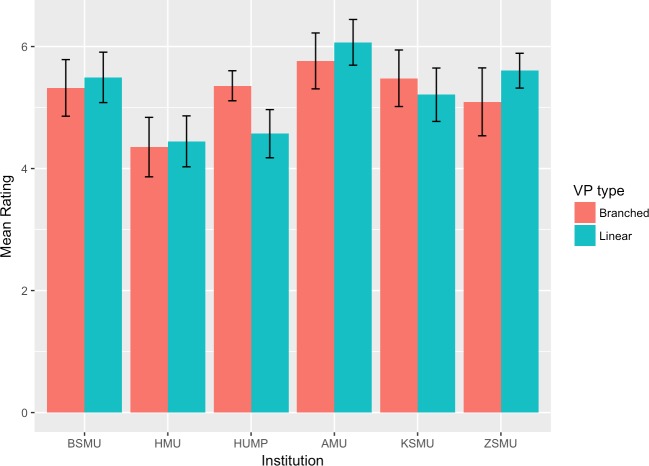
Help seeking mean ratings.

The mean scores and 95% confidence intervals for each group and institution are plotted in Figs 2–7. A table providing the list of calculated p values is provided in Table 3.

**Table 3:** Results of t-tests.

		*Motivation Scales*								*Learning Strategies*			
		*Value*		*Value*		*Expectancy*		*Expectancy*		*Cognitive and Metacognitive Strategies*		*Resource Management*	
Institution	VP Type	Intrinsic Goal Orientation		Task Value		Control of Learning Beliefs		Self-Efficacy for Learning and Performance		Critical Thinking		Help Seeking	
		Mean	P value	Mean	P value	Mean	P value	Mean	P value	Mean	P value	Mean	P value
BSMU	Linear	5.402	.750	5.507	.396	5.347	.337	5.526	.501	5.409	.496	5.494	.572
	Branched	5.483		5.724		5.069		5.375		5.566		5.322	
HMU	Linear	4.753	.314	4.948	.635	4.812	.678	4.092	.340	4.206	.199	4.462	.764
	Branched	5.037		5.069		4.944		4.343		4.525		4.351	
HUMP	Linear	4.738	.027	4.753	.016	4.848	.031	4.757	.070	4.717	. 015	4.571	.001
	Branched	5.286		5.275		5.360		5.198		5.284		5.357	
AMU	Linear	6.402	.324	6.477	.873	6.397	.848	6.375	.606	6.331	.553	6.069	.297
	Branched	6.609		6.510		6.440		6.483		6.460		5.764	
KSMU	Linear	6.370	.260	6.253	.806	5.519	.733	5.906	.540	5.911	.488	5.210	.387
	Branched	6.160		6.293		5.610		5.799		5.768		5.480	
ZSMU	Linear	6.625	.376	6.651	.675	6.234	.010	6.464	.001	5.831	.883	5.604	.103
	Branched	6.510		6.609		5.742		6.098		5.856		5.094	
All	Linear	5.718	.206	5.771	.146	5.534	.979	5.520	.675	5.393	.078	5.234	.973
	Branched	5.865		5.931		5.537		5.567		5.591		5.229	

The two-tailed unpaired t-tests comparing the linear vs branching VP design showed that in the majority of the subscales and institutions there was not a significant difference between the responses of the linear and the branching group. When the results for all institutions were aggregated and analysed as a single dataset, the results showed no significant differences between the linear and branched groups in any of the subscales. 5 of the 6 institutions did not report a significant difference between the linear and branching groups in Intrinsic Goal Observation, Task Value, Critical Thinking or Help Seeking, reporting p-values ranging from .103 to .883. In each of these subscales the exception was HUMP, which reported that the mean in the Branched group was significantly higher than that of the linear group, with p-values ranging from .001 to .031.

In contrast, both HUMP and ZSMU reported significant differences between the groups for the measure of Control of Learning Beliefs. HUMP again reported a significantly higher mean for the branching group (p = .031), but ZSMU reported a significantly higher mean for the linear group (p = .01). ZSMU was the only institution who reported a significant difference between the groups for Self-Efficacy of Learning Performance, with the mean for the linear group being higher than the branching (p = .001).

## Discussion

The results showed that, with a few exceptions at specific institutions, the type of VP did not make a significant difference to learner motivation. This demonstrates that those learner groups who received VPs with branched decision-making elements, which afforded the ability to make decisions and to subsequently be responsible for errors made, did not suffer any significant negative impact upon their learning motivation or perceived self-efficacy relating to the areas covered by the cases by the end of the six week period in which the cases were delivered. The generalisability of the study is addressed by using a repeated study design; by demonstrating a similar effect at multiple institutions we have shown that the findings are generalisable beyond the context of a specific institution.

Eva posited the idea that learners may improve their long-term performance as a result of errors induced in educational activities, but that there may be a consequential negative impact on learner confidence in the short term [7]. Simply put, “short term pain” leads to “long term gain”. We had hypothesised that the branching VPs may require more generative processing on behalf of learners–more “short term pain”. However, we have not found any evidence of this. Perhaps, by delivering a six week, six VP case exposure to these interventions, this “short term pain” has subsided.

The evidence provided by this study indicates that the length of exposure provided in this trial was sufficient to overcome any possible short-term negative impact to ratings of learner motivation and self-efficacy caused by the introduction of VPs with decision-making elements and supports our hypothesis to the extent that any negative impact was indeed “short term”. The additional cognitive load and generative processing demanded by including decision-making elements, as implied by the guiding activity principle [17], has thus been overcome by learners. Our expectation, informed by Mayer’s cognitive theory of multimedia learning [11] and evidence from similar implementations of decision-making [27], is that this generative processing should subsequently result in improved learning outcomes, although we did not attempt to measure levels of student performance in the scope of this study. Further research is required to test the impact on learning outcomes, along with the hypothesised negative impact to motivation and self-efficacy within the six week period of exposure to the intervention.

There were a few noted exceptions to the results that showed there was no significant difference between ratings provided by the branched and linear groups. Many of these differences were reported by HUMP, whose mean ratings were significantly different across all scales apart from Self-Efficacy of Learning and Performance. The only other significant differences were reported by ZSMU, who reported significant differences in the Control of Learning Beliefs and Self-Efficacy for Learning and Performance scales, scales which are both in the expectancy domain of the MSLQ. We noted that these exceptional results are from differing subscales, isolated in two institutions only, and that these institutions are from different countries thus are not using common translations of either the VP interventions or the survey instruments.

Our interpretation of the significant differences in the results from HUMP and ZSMU, aided by our repeated study design allowing for comparison between institutions, are that they potentially indicate the influence of existing institutional culture and expectations when attempting to measure the impact of curriculum interventions, and in particular those that relate to small-group teaching. Norman and Schmidt acknowledged that there is no such thing as a uniform intervention when considering the effectiveness of problem-based learning (PBL) curricula [56], while Maudsley describes the wide range of differing interpretations and models of PBL [57] as a specialised form of small group teaching. A huge range of different factors could potentially influence the perceptions and effectiveness of a small group intervention: an unbalanced group dynamic in which some voices are more dominant than others, a poor standard of tutoring in which the facilitator/tutor envisages themselves as a more didactic teacher, or even a lack of understanding about the learning process within the institution itself, thus encouraging a less than optimal experience. Evidence of this institutional effect may be supported by the ZSMU results particularly which, while exceptions to the general trend, were consistent within the value domain (as having no significant difference between linear and branched) and the expectancy domain (as having a significant difference). This indicates that some aspect of the training in that institution fostered higher expectancy values for those doing the linear cases than the branched, which is a local cultural effect unlike those in the other institutions. Further work, likely qualitative in nature and beyond the scope of this study, would be required to provide evidence to support and explain any institutional effect related to the quality of tutoring, group dynamics or learner expectancy.

We observed that, in general, the mean ratings reported for all sub-scales were higher for AMU, ZSMU and KSMU than for BSMU, HMU and HUMP. These groupings of institutions are not linked by a single translation of the VPs or the survey instruments to explain this difference. However, they are grouped by the fact that AMU, KSMU and ZSMU had previous experience of implementing PBL-style sessions using VPs as a consequence of their involvement in ePBLNet, a previous project that introduced a transformed PBL curriculum into these institutions [43,44]. In contrast, HMU, HUMP and BSMU had no prior experience of delivering small group sessions of this type, so the general difference in the level of mean ratings could potentially be explained by an institutional effect in which the adoption of a new style of learning takes some time to become culturally embedded within that institution. This is supported by anecdotal evidence emerging from discussions and observations at these institutions, but further formal research would be required to develop a fuller understanding of the complexity of the institutional cultural factors that impact upon this.

### Limitations

There are a number of limitations to the study that must be acknowledged. The populations at all participating institutions were drawn from undergraduate medical students at the same stage of their training. It does not necessarily hold that these findings would also be true of learners of different levels of expertise. Similar, the study populations at each institution were broadly homogenous in terms of gender and age; predominantly female and aged between 20 and 22. While this demographic balance was clearly representative across all institutions, this study cannot seek to understand how a change in this demographic (i.e. predominantly female, or older in age) would influence the results.

The study design randomised the allocation of teaching groups as clusters to the intervention, while ratings were reported as individuals and linked to the intervention that they received rather than the teaching group that they were in. As a consequence, we are unable to account for the impact of intra-class correlation within the teaching groups on the overall ratings, reducing the power of the study. The group-based nature of the intervention and the importance of the group dynamic in discussions means there was likely a strong sense of shared experience amongst learners in individual groups such that individual responses cannot be considered independent. Additionally, in a real-world setting there are a great range of hidden clustering effects that studies of this type cannot account for: gender, age, personality type, learning styles, and prior experience and education. However, the result of failing to adjust for non-independent responses is a calculation of standard error that is too small, and thus an increased likelihood of reporting a significant difference that does not really exist [58]. Since our findings show that there is no significant difference between the groups, we can conclude that any intra-class correlation is unlikely to have biased our results. The impact of any such effect is further mitigated by the fact that teaching within each group and institution was standardised; the same VP resources were used in identically laid out and equipped rooms, sessions were of a common duration and timetabled concurrently, and tutors were all trained directly by the TAME project using common instruction.

In studies of this type, in which participants are drawn from student populations and a study is run as part of the regular curriculum, we must also consider possible confirmation bias resulting from students anticipating what is expected of them, and reporting having higher motivation levels than they really had. If present, it would be anticipated that the extent of any confirmation bias would vary depending upon the institutional culture at different sites. However, in this study we would expect that this bias would have affected both groups equally, so is unlikely to strongly bias our results.

### Conclusions

The findings of this study demonstrated that the inclusion of decision-making elements into a series of VP interventions, designed to teach concepts of medical error over a period of six weeks, did not make a significant difference to undergraduate medical students’ motivation to engage with the learning activity, their perceived self-efficacy and understanding of the value of the learning, or their adopted learning strategies. These findings were mostly consistent across a range of institutions and regions, although there is evidence of an institutional effect and a need for a period of adaptation if an institution moves to small group and discursive methods of teaching from a more didactic curriculum.

The findings indicate that any negative impact upon a learner’s expectancy or perceptions of their performance, understanding of the value of the task resulting from the introduction of decision-making elements and the possibility of making errors into VP learning activities is short-term, and overcome within the six week period trialled here. Further work is required to identify whether the introduction of decision-making and errors into the intervention brings about a corresponding improvement in performance and subject-matter understanding, but existing studies have shown this might be anticipated.

## Supporting information

S1 File Original survey instrument (English). This supporting file provides the English language version of the original survey instrument in PDF format.
(PDF)

S1 Dataset Dataset of survey responses. This Excel spreadsheet provides the full set of survey responses upon which this study is based. The top row of the spreadsheet provides descriptive headers for the data. The question responses take a numerical value from 1 to 7, in which 1 corresponds to “Not at all true of me” and 7 corresponds to “Very true of me”.
(XLSX)
