# Supplementary material for: Virtual patients designed for training against medical error: Exploring the impact of decision-making on learner motivation
Source: PLoS One. 2019 Apr 23;14(4):e0215597. doi: 10.1371/journal.pone.0215597 (PMC6478293; doi:10.1371/journal.pone.0215597)
Supplement: S1 File — This supporting file provides the English language version of the original survey instrument in PDF format. (PDF) [file pone.0215597.s001.pdf]

TAME E1.2 - Learner Motivation Survey

Introduction

Thank you for agreeing to complete this survey, which aims to capture your experiences of participating in the teaching sessions using scenarios to introduce issues relating to medical error. You will be asked a series of questions about your approach to the scenarios, and the ways in which they may have motivated your learning.

The results from this survey will be used as part of a research project called 'Training in Medical Error'. By completing the survey you will be agreeing to your responses being used for this research. Your participation is voluntary, and you are free to withdraw at any time by not submitting the survey responses, without giving reason and without penalty. Your response will be anonymous, and the research team will not identify you by name in any reports using information obtained by this survey. By submitting the survey, you agree to the use of your comments being used as anonymous quotes in publications relating to this research.

* 1. Do you agree to the above terms? By clicking Yes, you consent that you are willing to answer the questions in this survey.

- ○ Yes
- ○ No

TAME E1.2 - Learner Motivation Survey

Your details

Please provide a few details about yourself.

2. What is your gender?

- ◯ Female
- ◯ Male

3. What is your age?

[ ]

4. What is the name of your institution?

- ◯ Hue University of Medicine and Pharmacy
- ◯ Hanoi Medical University
- ◯ Bukovinian State Medical University
- ◯ Zaporozhye State Medical University
- ◯ Karaganda State Medical University
- ◯ JSC Astana Medical University Kazakhstan

TAME E1.2 - Learner Motivation Survey

Your reactions to the case

5. Please rate the following based upon your feelings during and about the patient cases that you have gone through in these sessions?

	1 - Not at all true of me	2	3	4	5	6	7 - Very true of me
Whenever I read an assertion or conclusion in the scenarios I thought about possible alternatives.	○	○	○	○	○	○	○
Understanding the subject matter of these cases is very important to me.	○	○	○	○	○	○	○
I'm certain I can understand the most difficult material presented in the readings for these cases.	○	○	○	○	○	○	○
I'm certain I can master the skills being taught in these cases.	○	○	○	○	○	○	○
If I study in appropriate ways, then I will be able to learn the material in these cases.	○	○	○	○	○	○	○
I asked the tutor to clarify concepts I didn't understand well.	○	○	○	○	○	○	○
I often found myself questioning things I read in the scenarios to decide if I found them convincing.	○	○	○	○	○	○	○
When I couldn't understand the material in the scenarios, I asked another student in the class for help.	○	○	○	○	○	○	○
I tried to identify other students in the class whom I could ask for help if necessary.	○	○	○	○	○	○	○

	1 - Not at all true of me	2	3	4	5	6	7 - Very true of me
When working on the cases, I tried to relate the scenarios to material I already know.	○	○	○	○	○	○	○
I expect to do well in these cases.	○	○	○	○	○	○	○
I'm confident I can understand the most complex material presented in these cases.	○	○	○	○	○	○	○
Even if I was struggling with the scenarios, I tried to do the work on my own, without help from anyone.	○	○	○	○	○	○	○
I treated the cases as a starting point and tried to develop my own ideas about it.	○	○	○	○	○	○	○
When working on a patient scenario, I like when it really challenges me so I can learn new things.	○	○	○	○	○	○	○
If I don't understand the material in these cases, it is because I didn't try hard enough.	○	○	○	○	○	○	○
I like the subject matter of these cases.	○	○	○	○	○	○	○
I think I will be able to use what I learnt in these scenarios in other areas of my training.	○	○	○	○	○	○	○
Considering the difficulty of the cases, the tutor, and my skills, I think I will do well in these sort of scenarios.	○	○	○	○	○	○	○
The most satisfying thing for me in these cases was trying to understand the content as thoroughly as possible.	○	○	○	○	○	○	○
If assessed, I'm confident I could do an excellent job on the assignments and tests relating to these cases.	○	○	○	○	○	○	○

	1 - Not at all true of me	2	3	4	5	6	7 - Very true of me
When a theory, interpretation, or conclusion was presented in the scenarios I tried to decide if there was good supporting evidence.	○	○	○	○	○	○	○
If assessed, I believe I would receive an excellent grade in these cases.	○	○	○	○	○	○	○
It is important for me to learn the material in these scenarios	○	○	○	○	○	○	○
I think the material in these cases is useful for me to learn.	○	○	○	○	○	○	○
When I was choosing options in these cases, I sometimes chose the option that I felt I could learn from even if I thought it was incorrect.	○	○	○	○	○	○	○
I am very interested in the content area of these scenarios.	○	○	○	○	○	○	○
I tried to play around with ideas of my own related to what I was learning through the scenarios.	○	○	○	○	○	○	○
It is my own fault if I don't learn the material in these cases.	○	○	○	○	○	○	○
When working on a patient scenario, I prefer it to cover material that arouses my curiosity, even if it is difficult to learn.	○	○	○	○	○	○	○
If I try hard enough, then I will understand the material in these cases.	○	○	○	○	○	○	○
I'm confident I can understand the basic concepts taught in these cases.	○	○	○	○	○	○	○
